# A Multi-Objective Approach for Protein Structure Prediction Based on an Energy Model and Backbone Angle Preferences

**DOI:** 10.3390/ijms160715136

**Published:** 2015-07-03

**Authors:** Jyh-Jong Tsay, Shih-Chieh Su, Chin-Sheng Yu

**Affiliations:** 1 Department of Computer Science and Information Engineering, National Chung Cheng University, Min-Hsiung Township, Chia-yi County 62102, Taiwan; E-Mail: tsay@cs.ccu.edu.tw; 2 Department of Information Engineering and Computer Science, Feng Chia University, Taichung 40724, Taiwan

**Keywords:** backbone angle preferences, protein structure, multiobjective optimization, face-centered cubic

## Abstract

Protein structure prediction (PSP) is concerned with the prediction of protein tertiary structure from primary structure and is a challenging calculation problem. After decades of research effort, numerous solutions have been proposed for optimisation methods based on energy models. However, further investigation and improvement is still needed to increase the accuracy and similarity of structures. This study presents a novel backbone angle preference factor, which is one of the factors inducing protein folding. The proposed multiobjective optimisation approach simultaneously considers energy models and backbone angle preferences to solve the *ab initio* PSP. To prove the effectiveness of the multiobjective optimisation approach based on the energy models and backbone angle preferences, 75 amino acid sequences with lengths ranging from 22 to 88 amino acids were selected from the CB513 data set to be the benchmarks. The data sets were highly dissimilar, therefore indicating that they are meaningful. The experimental results showed that the root-mean-square deviation (RMSD) of the multiobjective optimization approach based on energy model and backbone angle preferences was superior to those of typical energy models, indicating that the proposed approach can facilitate the *ab initio* PSP.

## 1. Introduction

Protein structure prediction (PSP) is among the most challenging and unsolved research areas in biology. To date, most of the successful prediction methods have been designed to search for similar sequences in the Protein Data Bank (PDB) [1] for prediction, which is an approach named homology modelling [2]. However, this approach is inapplicable for dissimilar sequences. Therefore, other approaches are required, such as the *ab initio* modelling method. The greatest challenges confronting the *ab initio* method are the complexity of the configuration and the unknown factors of the folding mechanisms.

For the structural complexity issue, although the interaction between individual atoms can be calculated to model the folding of a protein in a search of the tertiary structure at the lowest free energy, the massive degree of computational complexity makes this approach infeasible. Therefore, researchers have proposed to develop simplified lattice models to reduce the computational complexity in modelling protein tertiary structure, such as a 2-D square [3,4,5], 2-D triangular [6], 3-D cubic [7,8,9], and 3-D face-centered cubic (FCC) [10,11,12,13,14] lattice models. Studies on these simplified models have typically used *C*_α_ atoms, which are centers of amino acids, as the backbone of the protein structure [15], and the research results have elucidated the relationship between protein sequences and structures.

Manuch and Gaur proposed a protein chain lattice fitting (PCLF) problem to investigate the similarity among various simplified discrete lattice models and protein structures in order to determine the applicability of the models to the protein structures, and proved that the problem was Non-deterministic Polynomial time (NP) complete [16]. Researchers have developed various PCLF tools, such as the LatFit tool [17] and LocalMove [18]. In particular, the LatFit tool provides numerous types of lattice models, including 2-D square, 3-D cubic, FCC, and 210-type models. The LatFit tool can be used to identify the lattice structure that is most similar to the native structure in the lattice models, and it provides a visual comparison of results and root-mean-square deviation (RMSD) values. Therefore, the LatFit tool is a preferable research instrument.

Mann *et al.* [19] used the LatFit tool to evaluate the applicability of various lattices and the accuracy of structure representations achieved using backbone-only [15] and backbone-side-chain models [20]. They concluded that the FCC lattice was the preferable lattice model, and the *C*_α_-centered backbone-only model was superior for protein representation. Therefore, this study used a 3-D FCC lattice model and backbone representation to perform an experimental analysis.

In terms of unknown factors of the folding mechanisms, according to the laws of thermodynamics, the native structure of proteins is typically in lowest free energy state [21]. Known factors that drive protein folding into native structures involve (i) hydrogen bonds; (ii) van der Waals interactions; (iii) backbone angle preferences; (iv) electrostatic interactions; (v) hydrophobic interactions; and (vi) chain entropy [2]. Among these factors, hydrophobic interactions or the interactions among amino acids are most commonly adopted factors, such as the hydrophobic-polar (HP) model [15], Barrera matrix [22], and Miyazawa–Jernigan (MJ) matrix [23], all of which are based on contact-based statistical energy models aimed at guiding various calculation methods to determine the global optimal solution: to find the structure with the lowest free energy.

However, this type of energy model for single-objective optimisation can achieve only a limited level of success. In terms of accuracy, a gap remains between the prediction results achieved by this type of energy model and the native structure. This study focused on another factor that also triggers protein folding: backbone angle preferences. Considering backbone angle preferences is necessary. Previous studies have shown that dihedral angles are formed between the peptide plane and *C*_α_ in amino acids, namely, phi (Φ) and psi (ψ) angles, which determine the backbone structure of proteins. In most proteins, the angle combination (Φ, ψ) is located within a fixed area of the Ramachandran plot [24]. In other words, when an energy model is used as an objective function, a compact globular structure is typically formed to obtain the maximal contact energy (*i.e.*, the lowest free energy). However, in this type of compact protein structure the dihedral angles may be neglected and may generate unreasonable conformations, such as overly small folding angles or segment structures in a form that cannot possibly exist.

Adding the factor of backbone angle preferences generated a bi-objective optimisation problem to the original energy model. When an optimisation problem is related to multiple objectives, the task of searching for the optimal solution is referred to as an multiobjective optimisation problem. Previous studies have proposed numerous multiobjective optimisation methods, such as the nondominated sorting genetic algorithm (NSGA) [25] and NSGA-II [26]. These are superior solutions for bi- or tri-objective optimisation problems and were therefore adopted by this study to investigate whether the proposed multiobjective model and method based on the energy model and backbone angle preferences can enhance the PSP accuracy.

Finally, the experimental results showed that the structural similarity in the proposed multiobjective methods based on energy models and backbone angle preferences were superior to the traditional energy models. The result proves that the proposed method is an effective *ab initio* method that is applicable to the PSP problem

## 2. Results and Discussion

The present study reports a series of experiments to evaluate the proposed methods on the multi-objective on the protein structure prediction problem. Seventy-five amino acid sequences with lengths ranging from 22 to 88 amino acids were selected from the CB513 data set to be the benchmarks.

**Figure 1:**
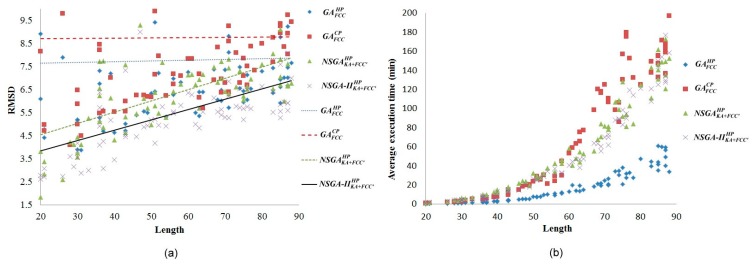
(**a**) Root-mean-square deviation (RMSD) comparison among four objective functions; (**b**) Comparison of running time complexity among four objective functions.

Because of the numerous types of energy models examined in this study, the experiments were conducted in two stages to simplify the problem. Stage 1: the HP [15] and Berrera [22] energy models were first used for the experiment conducted on all of the data sets, and the appropriate energy model was selected as the objective function. The objective function with another objective function formed a multiobjective optimization problem. The objective functions are defined in Equations (1) and (2); Stage 2: this study proposed two improved multiobjective optimisation methods ($NSGA_{KA+FCC^{\prime }}^{HP}$ and $NSGA-II_{KA+FCC^{\prime }}^{HP}$) incorporating rotation-based crossovers, local search of generalized pull move and *K*-site mutation methods [14]. Multiobjective optimisation experiments were conducted on all benchmarks. Figure 1 shows the experimental results of the proposed single-objective and multiobjective optimisation methods.

In terms of single-objective structure prediction topic, in most of the data sets the RMSD values in the $GA_{FCC}^{HP}$ energy model were superior to that in the $GA_{FCC}^{CP}$ energy model (Figure 1a). Additionally, the time complexity of the $GA_{FCC}^{HP}$ energy model was comparatively lower (Figure 1b). Thus, the $GA_{FCC}^{HP}$ fitness function was used as one of the objective functions.

In terms of multiobjective structure prediction topics, Figure 1a shows the experimental result of the proposed multiobjective optimisation methods. Both the $NSGA_{KA+FCC^{\prime }}^{HP}$ and $NSGA-II_{KA+FCC^{\prime }}^{HP}$, which were based on both energy models and backbone angle preferences, were superior to the $GA_{FCC}^{HP}$ energy models, which were based only on energy models, indicating that the proposed energy model and backbone angle preference-based multiobjective methods effectively improved the PSP accuracy. $NSGA-II_{KA+FCC^{\prime }}^{HP}$ was particularly optimal. However, finding the optimization in a lattice model is an NP-hard problem [27] which is why both the $NSGA_{KA+FCC^{\prime }}^{HP}$ and $NSGA-II_{KA+FCC^{\prime }}^{HP}$ exhibited an exponential growth in time complexity. This indicates that the effectiveness of the two methods could be improved further (Figure 1b). Nevertheless, compared to the off-lattice method [28,29,30], the running time is short; we can get the best approximate solution within an acceptable running time.

### 2.1. Comparisons with Visualization

The present study compares three models in terms of visual comparison, and are summarized in Table 1, which shows that the HP energy model prediction results exhibited a typical hydrophobic core structure; some of which exhibited globular structures. However, the tertiary structure of the proteins did not necessarily exhibit a globular conformation. For example, the tertiary structure of the proteins in Table 1, items 2 and 6, are in helical conformation formed by an alpha-helix, implying that the tertiary structure may be influenced by unknown folding mechanisms. The visual comparisons in Table 1 show that the prediction results of the $NSGA-II_{KA+FCC^{\prime }}^{HP}$ were similar to the native structure.

**Table 1:** Three different types of energy model for visual comparison.

No.	PDB-ID	Native	$GA_{FCC}^{HP}$	$NSGA_{KA+FCC^{\prime }}^{HP}$	$NSGAII_{KA+FCC^{\prime }}^{HP}$
01	1EDN Len.21	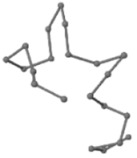	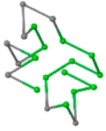	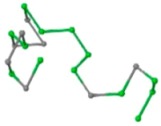	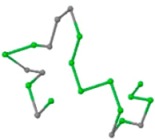
02	1COI Len.29	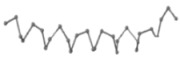	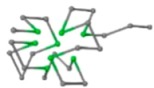	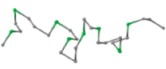	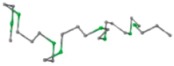
03	1MRT Len.31	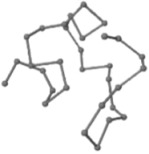	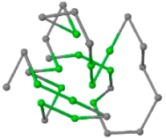	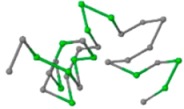	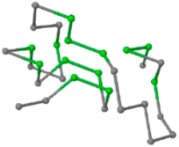
04	2ERL Len.40	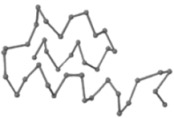	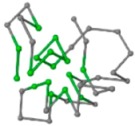	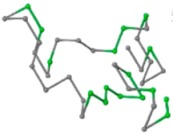	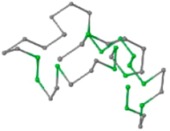
05	1CRN Len.46	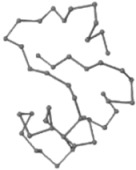	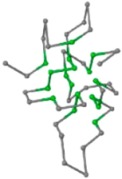	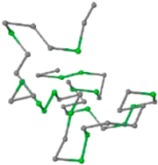	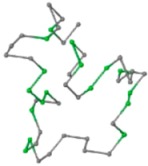
06	1RPO Len.61	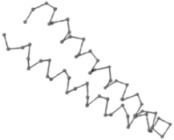	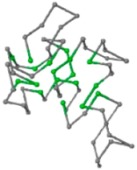	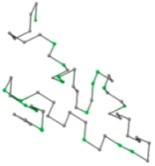	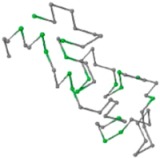

In the figure green balls indicate hydrophobic amino acids while the grey balls indicate the hydrophilic amino acids. The results were input into the HPview tool (http://cpsp.informatik.uni-freiburg.de:8080/ViewJSP.jsp) to obtain the backbone structure; PDB-ID: A 4-character PDB identification (ID) code is assigned to each new structure at the time of deposition. They serve as the unique, immutable identifier of each entry in the Protein Data Bank.

The experiments were repeated 30 times for each benchmark. Table S1 shows the optimal experimental results of the numerical values of all data sets. The experimental results show that using both the $NSGA_{KA+FCC^{\prime }}^{HP}$ and $NSGA-II_{KA+FCC^{\prime }}^{HP}$ methods for prediction can increase the structural accuracy after considering the factor of the backbone angle preferences. In particular, the $NSGA-II_{KA+FCC^{\prime }}^{HP}$ yielded a mean RMSD of 5.5 Å for the 75 benchmarks, which was markedly lower than that generated by the widely adopted HP model (7.76 Å), indicating the novelty of the proposed method. Consequently, the visual comparison and the comparison of RMSD yielded identical results. These results are to be expected, because the multiobjective method obtains a set of pareto-optimal front solutions, but also because the solution set of this group has been included in a single-objective solution. Thus, the proposed multiobjective optimisation method can enhance structure simulation accuracy for *ab initio* PSP.

### 2.2. Comparisons with Off-Lattice Models

An off-lattice model is a continuous model that has a higher resolution of conformational representations not limited by the constraints. These methods will increase the conformation search space. In addition, the design of folding mechanisms still remains an unresolved issue [28,29,30]. Quark [28] is a state-of-the-art method in off-lattice modelling. The Quark energy function covers three hierarchies packaging: atom-, residue- and topological level energy. It is demonstrated that the short protein (<100 residues) can give a successful outcome.

**Figure 2:**
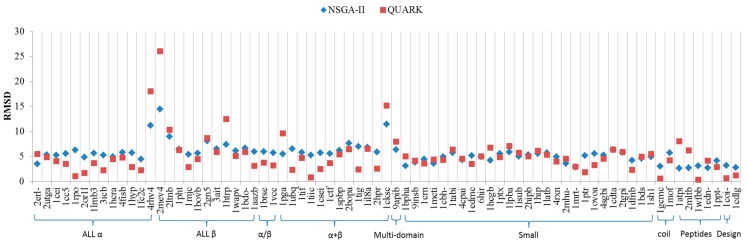
Comparison of the proposed approach with the QUARK.

Figure 2 shows the comparison with Quark results. According to Figure 2 the Quark in All α, All β, α and β (a/b), and α and β (a + b) class perform better in structure similarity presented in this study by NSGA-II. However, the prediction in Multi-domain, Small proteins, Coiled coil, and the like Peptides class resulted from NSGA-II works slightly better than Quark. In addition, compared with Quark webserver running time, NSGA-II takes only about 1/3 of the running time (not including hardware equipment considerations).

## 3. Materials and Methods

### 3.1. Materials

In this section, we review the 3D FCC lattice, (κ, α)-pair angle preferences, fitness function and RMSD which are used in our approach.

#### 3.1.1. 3D FCC Lattice

**Figure 3:**
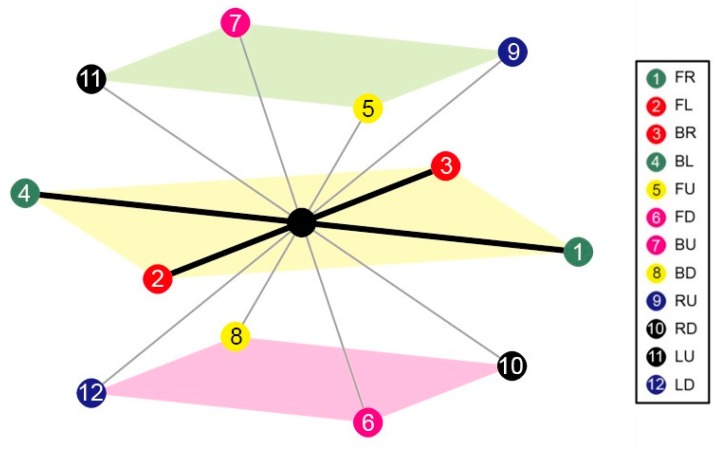
The FCC lattice model: Each lattice point has 12 neighbours schematic.

In FCC lattice, There are 12 neighbours of each lattice point which are labelled as numbers from 1 to 12, where 1 is for *FL* (+1, +1, 0), 2 for *FR* (+1, −1, 0), 3 for *FU* (−1, +1, 0), 4 for *FD* (−1, −1, 0), 5 for *BL* (+1, 0, +1), 6 for *BR* (+1, 0, +1), 7 for *BU* (−1, 0, +1), 8 for *BD* (−1, +0, −1), 9 for *LU* (+0, +1, +1), 10 for *LD* (+0, +1, −1), 11 for *RU* (+0, −1, +1), and 12 for *RD* (+0, −1, −1). Symbols *FL*, *FR*, *FU*, *FD*, *BL*, *BR*, *BU*, *BD*, *LU*, *LD*, *RU* and *RD* are used to denote fold directions with *FL* for front-left, *FR* for front-right, *FU* for front-up, *FD* for front-down, *BL* for back-left, *BR* for back-right, *BU* for back-up, *BD* for back-down, *LU* for left-up, *LD* for left-down, *RU* for right-up and *RD* for right-down. The vector following each symbol is its corresponding direction vector. Consequently, a 3D FCC model was proposed and developed as shown in Figure 3.

#### 3.1.2. (κ, α)-Pair Angle Preferences

**Figure 4:**
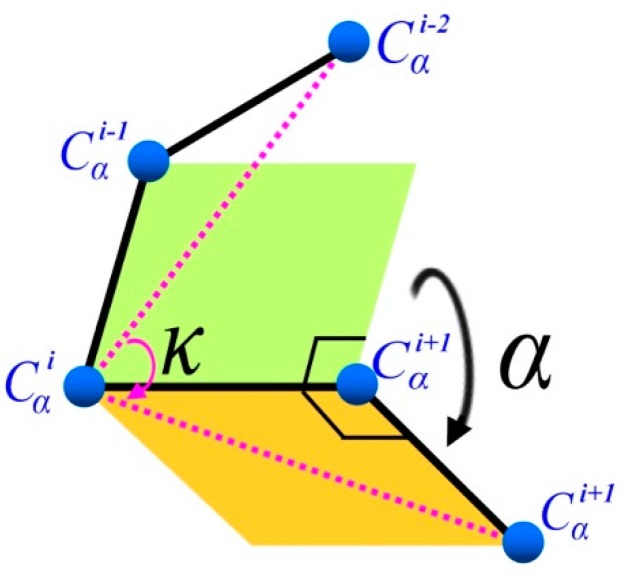
(κ, α) pair backbone angle schematic.

κ angle is defined by the angle formed by three *C*_α_ atoms of residues *i* − 2, *i*, and *i* + 2, ranging from 0° to 180°. α angle, on the other hand, is defined by the angle between two planes formed by four *C*_α_ atoms of residues *i* − 1, *i*, *i* + 1 and *i* + 2, ranging from −180° to 180° as shown in Figure 4 [31]. In order to understand the (κ, α) angle ranging of FCC lattice, which is a special discrete space, an enumeration method was used in our study to calculate all possible (κ, α)-pair angles. Our results show that the κ angle was from 30° to 150° and α angle was within 60°, 80°, 180°, −130°, −110° and −10° of six discrete spaces. In this study, (κ, α) angle constraints are used to ensure the structural rationality of fragments.

Subsequently 9083 protein sequences were selected from PISCE [32] and their structural similarity is lower than 30%, but with a higher structure resolution (*R* factor < 1 & resolution < 2.5). Afterwards (κ, α) pair angle preferences were calculated by processing via DSSP algorithm [33] and a (κ, α) pair angle preferences matrix that is suitable in FCC lattice was proposed as shown in Table 2.

**Table 2:** (κ, α) pair backbone angle preferences matrix.

	α Angle						
**κ Angle**		**60°**	**80°**	**180°**	**−130°**	**−110°**	**−10°**
	**30°**	0	0	0	0	0	0
	**40°**	0	0.05	0.01	0	0	0
	**50°**	0.21	0.73	0.18	0.06	0	0.05
	**60°**	1.24	1.5	0.34	0.49	0	0.38
	**70°**	17.87	1.76	0.14	0.55	0	0.95
	**80°**	11.08	0.59	0.21	0.51	0.02	1.65
	**90°**	1.28	0.58	0.31	0.64	0.25	2.44
	**100°**	0.87	0.68	0.39	0.98	0.56	2.98
	**110°**	0.72	0.70	0.47	1.29	0.96	2.14
	**120°**	0.28	0.56	0.57	1.22	1.78	1.04
	**130°**	0.08	0.24	0.47	1.39	2.08	0.57
	**140°**	0.04	0.10	0.40	1.99	2.03	0.43
	**150°**	0.02	0.02	0.21	7.43	17.46	0.60

60° probability value represent the sum of DSSP value ranging from 130° to 140°, 80° represent that from 150° to 190°, 180° represent that from 200° to 260°, −130° represent that from −100° to −50°, −110° represent that from −40° to −10° and 10° to 40°, −10° represent that from 50° to 120°.

#### 3.1.3. Fitness Function

In order to understand the effect of (κ, α) pair angle preferences on protein structure, the (κ, α) pair angle preferences matrix was defined as the fitness function and represented as $K^{KA}$. According to the lowest free energy assumption, (κ, α) free energy in this study was multiplied by −1 to fit into the assumption. Let $s=s_{1}s_{2}\cdots s_{n}$ be an amino acid sequence, and $c=p_{1}p_{2}\cdots p_{n}$ be a valid conformation for *s*. Then the κ and α free energy *E*(*c*) of *c* is defined as follows:

(1) $$K^{KA}\left(c\right)=\;-\sum_{i=3}^{n-2}\sum_{j=i+2}^{n}contact\;\left(p_{i},p_{j}\right)$$

According to the contact-based statistical energy model, the fitness functions were denoted as *E^HP^* and *E^CP^*. The fitness function *E^HP^* in Equation (2) signifies that the HP model calculates only the energy that exists in the interaction among the hydrophobic amino acids [15]. The fitness function *E^CP^* in Equation (3) represents all energy models for the interaction among the 20 amino acids. This study groups amino acids such as C, F, I, L, M, V, W and Y into hydrophobic (represented by H) and amino acids such as H, A, T, G, P, S, Q, R, N, D, E and K into polar (represented by P).

(2) $$E^{HP}\left(c\right)=\;-\sum_{i=1}^{n-2}\sum_{j=i+2}^{n}contact\;\left(p_{i},p_{j}\right)$$

(3) $$E^{CP}\left(c\right)=\;-\sum_{i=1}^{n-2}\sum_{j=i+2}^{n}contact\;\left(p_{i},p_{j}\right)$$

#### 3.1.4. Root-Mean-Square-Deviation

Root mean square deviation is one of the most used instruments for structure comparison. This study uses RMSD to evaluate protein structure similarity. Although it is an imperfect structure comparison method and the interpretation of the RMSD is not clear [30,34], the numerical results still have some significance. To normalise the data, the neighbourhood distance ($\sqrt{2}$) in the 3-D FCC lattice model was set to 3.8 Å to indicate the mean distance between two continuous *C*_α_ in real proteins. The RMSD is defined as follows [35]:

(4) $$RMSD=\;\sqrt{\frac{\sum_{1<i<j<n}{\left(c_{i,j}^{model}-c_{i,j}^{real}\right)}^{2}}{\left(\begin{array}{c} n\\ 2 \end{array}\right)}}$$

### 3.2. Methods

This study proposed a novel energy model- and backbone angle preference-based multiobjective model for the PSP problem. The proposed model features the advantages of the protein hydrophobic cores and characteristics of the backbone angle preferences in the protein structures. In multiobjective optimisation problems, the solution is not an optimal single-objective solution, but a trade-off solution set that fulfils all objectives, which is also named Pareto-optimal front solution set. Each solution at the Pareto front is not dominated by other solutions and is hence called a nondominated solution. In other words, in general situations where a problem involves multiple objectives, no single optimal solution exists; rather, a set of optimal solutions exist, namely the Pareto-optimal front solution set. To solve this multiobjective optimisation problem, this study proposed a multiobjective method and demonstrated its performance by comparing it with the single-objective model.

For the single-objective model, this study integrated two common energy models into the problem solving process, namely, the hydrophobic-polar (HP) and contact potential (CP) energy models. The terms $GA_{FCC}^{HP}$ and $\;GA_{FCC}^{CP}$ were used to express the free energy (HP or CP) protein structure, which was determined using genetic algorithms. The superscripts “HP” and “CP” are used to indicate whether the energy models used HP or CP, respectively, of which the free energy was calculated using the corresponding Equations (1) and (2). The subscript “FCC” indicates that the lattice model was used.

In the multiobjective model, κ–α angle preferences and angle constraint, denoted by $NSGA_{KA+FCC^{\prime }}^{HP}$ and $NSGA-II_{KA+FCC^{\prime }}^{HP}$, were incorporated into the aforementioned energy models. NSGA [25] and NSGA-II [26] are two commonly used multiobjective optimization methods that are suitable for solving optimization problems involving two or three objectives. The subscript “KA” denotes the κ–α free energy (Equation (3)), and “FCC*'*” indicates the added κ–α angle constraint. Candidate solutions that do not meet the requirements of the κ–α angle preferences and angle constraint were discarded.

The process of evolution algorithm is realised through the mechanisms of selection (parent and survival selection) and reproduction (crossover and mutation). Parent selection is the process of collecting chromosomes to be selected as parents for crossover. These studies apply tournament selection method in which the better of two randomly selected chromosomes is selected as one parent. Survival selection refers to the “survival of the fittest”, which is a clear survival mechanism that decides which chromosomes are inherited by the next generation. Specifically, chromosomes are ranked according to their fitness value, and those that are ranked highly are considered suitable for the environment and have a high probability of survival. In the $NSGA_{KA+FCC^{\prime }}^{HP}$ method, chromosomes are selected based on the dominance rank, whereas those in the $NSGA-II_{KA+FCC^{\prime }}^{HP}$ method are selected based on their dominance rank and crowding distance.

#### 3.2.1. Crossovers Operate

**Figure 5:**
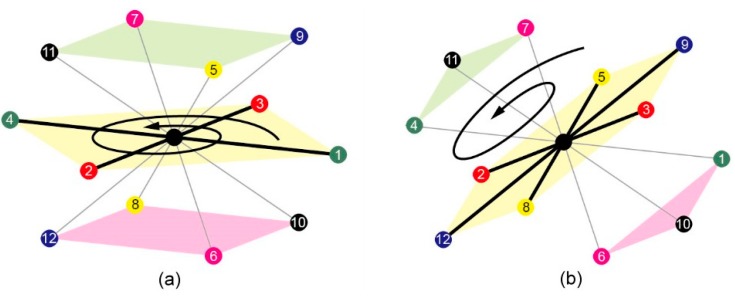
(**a**) Square-based rotation schematic; and (**b**) Triangle-hexagon-based rotation schematic.

To increase the crossover success rate, rotation-based crossovers [14] were adopted in this study. For this method, the protein backbone structure in the FCC lattice was regarded as a rigid structure, in which each carbon atom in an amino acid represents a coordinate point in the FCC lattice, and the distance between the coordinate points is fixed. Thus, before the rotation, point *G* was first selected from the rigid structure to represent the entire protein structure, and this point was set as the center of the structure. Subsequently, fixed axis rotation was performed on a vertical axis intersecting *G*. After the structure was rotated, the coordinate values of random coordinate points, denoted as *P*, can be rebuilt only after determining the relative positions of *P* and *G*. Thus, in this study, the rotation-based crossover operation began with randomly selecting a coordinate point as the center of the protein structure (one-point crossover). Subsequently, the rigid structure was subjected to square-based rotation Figure 5a and triangle-hexagon-based rotation Figure 5b. In square-based rotation, each plane contains four coordinate points that rotate around the center point of structure on three vertical axes (*x*, *y*, *z*). The rotational angles are π/2, π, 3π/2. Fixed axis rotation was performed to produce nine offspring (3 vertical axes × 3 rotational angles). In triangle-hexagon-based rotation, the top and bottom planes are triangles formed by three coordinate points; the plane in the middle is a hexagon formed by six coordinate points that rotate around the center point of structure on four vertical axes. The rotational angles for the four axes are 2π/3 and 4π/3. Fixed axis rotation was performed to produce eight offspring (four vertical axes × two rotational angles). Thus, each rotation-based crossover will generate at most 17 new chromosomes. Each rotation is performed by first partitioning all lattice points into parallel planes, and then rotating all planes synchronously. After a complete rotation, the parents’ conformation can be preserved, the success rate of crossover can be increased and the range of local search can be extended.

#### 3.2.2. Local Search

In order to improve the offspring we used a generalized pull move (GPM) [14] method that searches and examines iteratively the set of points in a neighbourhood of the current solution and replaces the current solution with a better existing neighbour. For the GPM operation, two connected coordinate points ($P_{i}$, $P_{i+1}$) were randomly selected from the protein structure. A previously unused neighbour point in the lattice was selected as the candidate point ($P_{i+1}^{\prime }$). The second coordinate point ($P_{i+1}$) was then moved towards the candidate point ($P_{i+1}\to P_{i+1}^{\prime }$). In cases where there was more than one candidate point, one of them was selected randomly. Subsequently, the remaining points ($P_{i+2,\;\ldots ,\;}P_{i+n}$) were moved towards the candidate point by applying the step for constructing a new structure.

#### 3.2.3. Mutations Operate

A *K*-site mutation method [14] was adopted to enable mutation operations to search for the optimal conformation by moving *K* consecutive points in the conformation. This method can avoid drastic changes that destroy the global structure of the current configuration.

#### 3.2.4. Termination

The process repeated a fixed number of iteration times. When terminated, the best conformation remaining in the population is returned. Throughout the reproduction stage, a self-avoiding walk (SAW) and the (κ, α)-pair angle constraints were satisfied, thereby ensuring the legitimacy of the conformation, and the fitness values of the energy models and (κ, α) angle preferences were computed. Illegitimate conformations were discarded.

#### 3.2.5. Data Set

To prove the applicability of the proposed energy model- and backbone angle preference-based multiobjective model to the PSP problem, 75 amino acid sequences with lengths ranging from 22 to 88 amino acids were selected from the CB513 data set for use as benchmarks [36]. The PDB IDs of the 75 data sets are listed as follows:

*1atpi*, *1cdlg*, *1edn-*, *1bpha*, *2mltb*, *1mcti*, *1coi-*, *9insb*, *2mhu-*, *1dfnb*, *1mrt-*, *1gcmc*, *1cbh-*, *1ppt-*, *1tabi*, *1tiic*, *9apib*, *1wfbb*, *4cpai*, *1edmc*, *4rhv4*, *2erl-*, *1fc2c*, *1htrp*, *1bds*, *1crn*, *2ltnb*, *1sh1*, *6hir*, *1ptr*, *4sgbi*, *1hcgb*, *1hcra*, *1mof*, *4rxn*, *1ovoa*, *1pga*, *2mev4*, *2tgpi*, *1cdta*, *1rpo*, *1isub*, *1csei*, *2or1l*, *1ptx*, *1wapv*, *1ctf*, *1mjc*, *1bovb*, *2utga*, *1il8a*, *2hipb*, *1spbp*, *4fisb*, *1latb*, *3ait*, *1hyp*, *3icb*, *1ubq*, *1tif*, *1vcc*, *1cksc*, *1bdo-*, *1cc5*, *1pht*, *1lpba*, *2bopa*, *1cei*, *1hip*, *1brse*, *2hpr*, *1aazb*, *2gn5*, *1lmb3*, *1tig.*

#### 3.2.6. Experimental Parameters

Regarding the parameter setting, because the sequence lengths in data sets varied, the population size was set to the sequence length, and the number of iterations was set to double the length of the protein sequence. Table 3 lists the parameters for the adopted single-objective or multi-objective evolutionary algorithms based on $GA_{FCC}^{HP}$, $NSGA_{KA+FCC^{\prime }}^{HP}$ and $NSGA-II_{KA+FCC^{\prime }}^{HP}$.

**Table 3:** Parameter setting for the four evolutionary algorithms used in experiments.

Operations/Parameters	Setting
Representation	1–12 Represents 12 vertex coordinates
Population size	Equal to substring_length
Selection	Tournament selection
Crossover rate *P*_c_	0.85
Mutation rate *P*_m_	1/(substring_length)
*K* size	3
Termination	Substring_length *2 generations

## 4. Conclusions

The *ab initio* PSP is an open and unsolved problem. Various factors may drive proteins to fold into a native structure; however, how amino acid sequences fold into a tertiary structure remains unknown. After decades of research efforts, the single-objective energy model optimisation problem has been solved, although the structural accuracy can be improved further. Therefore, this study proposed a novel multiobjective optimisation model and method based on the energy model, (κ, α)-pair backbone angle preferences and constraint in order to enhance the structure simulation accuracy.

The experimental results showed that using both the $NSGA_{KA+FCC^{\prime }}^{HP}$ and $NSGA-II_{KA+FCC^{\prime }}^{HP}$ methods for prediction can increase the structural accuracy after considering the factor of the backbone angle preferences. According to prediction of the results of classification as showed in this study our method is suitable for Multi-domain, Small proteins, Coiled coil, and the like Peptides class prediction. Thus, the proposed multiobjective optimisation method can enhance the *ab initio* PSP structure simulation accuracy.

## Supplementary Materials

Supplementary materials can be found at http://www.mdpi.com/1422-0067/16/07/15136/s1.
