# Supplementary material for: A Multi-Objective Approach for Protein Structure Prediction Based on an Energy Model and Backbone Angle Preferences
Source: Int J Mol Sci. 2015 Jul 3;16(7):15136–49. doi: 10.3390/ijms160715136 (PMC4519891; doi:10.3390/ijms160715136)
Supplement: Supplementary file 1 [file ijms-16-15136-s001.pdf]

Supplementary Information

Table S1. Comparison three different types of energy model with the QUARK.

No.	SCOP	PDB-ID	Length	GA_{FCC}^{HP}	$NSGA_{KA+FCC'}^{HP}$	$NSGAII_{KA+FCC'}^{HP}$	QUARK
**01**		**2erl-**	**40**	**4.64**	**4.32**	**3.46**	**5.47**
02		2utga	70	7.35	6.42	5.29	**4.81**
03		1cei	85	6.62	6.72	5.27	**4.05**
04		1cc5	83	6.35	6.53	5.52	**3.52**
05		1rpo	61	10.58	7.16	6.29	**1.02**
06	**All α**	2or11	63	5.36	5.69	4.84	**1.67**
07		1lmb3	87	7.01	6.69	5.61	**3.66**
08		3icb	75	5.97	6.23	5.2	**2.22**
09		1hcra	52	7.22	5.47	4.95	**4.41**
10		4fisb	73	10.81	6.61	5.77	**4.79**
11		1hyp	75	6.43	6.06	5.69	**2.89**
12		1fc2c	43	5.01	4.64	4.45	**2.21**
13		4rhv4	40	19.83	12.9	**11.2**	18.03
14		2mev4	58	28.1	**10.72**	14.4	25.99
15		2ltnb	47	12.67	9.29	**9**	10.34
16		1pht	83	7.44	8.06	6.42	**6.24**
17		1mjc	69	6.01	7.71	5.39	**2.89**
18	**All β**	1bovb	69	6.49	6.98	5.65	**4.47**
19		2gn5	87	9.24	**7.61**	8.08	8.68
20		3ait	74	7.47	7.32	6.53	**5.88**
21		1htrp	43	15.36	7.59	**7.34**	12.44
22		1wapv	67	7.37	7.47	6.14	**5.08**
23		1bdo-	80	7.3	7.59	6.63	**5.86**
24	**α and β (a/b)**	1aazb	87	7.45	6.7	5.92	**3.13**
25		1brse	86	7.02	6	5.95	**3.73**
26		1vcc	77	6.54	6.96	5.69	**3.15**
27		1pga	56	6.28	6.42	**5.48**	9.63
28		1ubq	76	7.14	6.64	6.49	**2.34**
29		1tif	76	6.07	6.51	5.76	**4.68**
30		1tiic	36	15.13	6.23	5.2	**0.79**
31		1csei	63	6.39	6.93	5.69	**2.44**
32	**α and β (a + b)**	1ctf	68	6.06	6.82	5.53	**3.64**
33		1spbp	71	8.11	7.43	6.21	**5.36**
34		2bopa	85	8.77	9.11	7.61	**6.45**
35		1tig	88	7.65	6.77	6.99	**2.37**
36		1il8a	71	8.82	7.81	6.86	**6.48**
37		2hpr	87	6.68	6.65	5.91	**2.53**
38		1cksc	78	16.31	**10.74**	11.42	15.14

Table S1. *Cont.*

No.	SCOP	PDB-ID	Length	GA^{HP}_{FCC}	$NSGA^{HP}_{KA+FCC'}$	$NSGAII^{HP}_{KA+FCC'}$	QUARK
39	**Multi-domain**	9apib	36	10.59	7.71	**6.38**	7.92
40		1bpha	21	4.78	3.37	**3.09**	5.01
41		9insb	30	5.2	3.57	**3.84**	4.14
42		1crn	46	5.4	5.45	4.46	3.54
43		1mcti	28	4.16	4.22	**3.6**	4.34
44		1cbh-	36	5.28	5.11	4.91	**4.30**
45		1tabi	36	6.75	6.55	**5.72**	6.35
46		4cpai	37	4.7	4.87	**4.26**	4.52
47		1edmc	39	7.2	6.13	5.17	**3.52**
48		6hir	49	5.51	5.7	**4.94**	5.02
49		1hcgb	51	9.41	5.8	**4.18**	6.73
50		1ptx	64	6.39	7.16	5.54	**4.81**
51		1lpba	85	6.94	7.82	**5.85**	7.06
52		1isub	62	5.49	6.71	**5.01**	5.70
53	**Small proteins**	2hipb	71	5.71	6.32	5.29	**4.98**
54		1hip	85	5.92	7.06	5.56	6.10
55		1latb	74	6.57	7.28	5.72	5.29
56		4rxn	54	5.32	5.29	4.92	**3.97**
57		2mhu-	30	3.9	3.77	**3.56**	4.50
58		1mrt-	31	3.88	4.13	**2.87**	**2.94**
59		1ptr	50	6.33	4.98	5.18	**1.83**
60		1ovoa	56	6.98	5.95	5.54	**3.28**
61		4sgbi	51	6.41	6.37	5.21	**4.53**
62		1cdta	60	7.25	7.14	6.35	6.34
63		2tgpi	58	6.81	6.74	5.82	5.88
64		1dfnb	30	5.19	4.43	4.19	**2.34**
65		1bds	43	4.73	5.33	**4.57**	4.90
66		1sh1	48	5.56	6.12	**4.89**	5.46
67	**Coiled coil**	1gcmc	33	12.97	5.27	3	**0.58**
68		1mof	53	14.28	7.68	5.75	4.21
69		1atpi	20	8.92	3.83	**2.62**	8.01
70		2mltb	26	7.9	2.6	**2.74**	6.17
71	**Peptides**	1wfbb	37	5.61	7.73	3.07	**0.32**
72		1edn-	21	4.42	2.84	**2.71**	4.10
73		1ppt-	36	7.32	5.12	4.09	2.83
74	**Designed**	1coi-	29	10.95	4.12	3.16	**0.67**
75		1cdlg	20	6.11	1.84	2.77	**1.19**
	Average of RMSD			**7.76**	**6.34**	**5.5**	**5.09**

Words in bold indicate the lowest RMSD.
